# Evolution of the *Piscine orthoreovirus* Genome Linked to Emergence of Heart and Skeletal Muscle Inflammation in Farmed Atlantic Salmon (*Salmo salar*)

**DOI:** 10.3390/v11050465

**Published:** 2019-05-22

**Authors:** Kannimuthu Dhamotharan, Torstein Tengs, Øystein Wessel, Stine Braaen, Ingvild B. Nyman, Elisabeth F. Hansen, Debes H. Christiansen, Maria K. Dahle, Espen Rimstad, Turhan Markussen

**Affiliations:** 1 Faculty of Veterinary Medicine, Norwegian University of Life Sciences, 0454 Oslo, Norway; dhamokan@nmbu.no (K.D.); oystein.wessel@nmbu.no (Ø.W.); stine.braaen@nmbu.no (S.B.); ingvild.nyman@nmbu.no (I.B.N.); elisabeth.hansen@nmbu.no (E.F.H.); turhan.markussen@nmbu.no (T.M.); 2 Faculty of Chemistry, Biotechnology and Food Science, Norwegian University of Life Sciences, 1433 Ås, Norway; torstein.tengs@nmbu.no; 3 Faroese Food and Veterinary Authority, National Reference Laboratory for Fish Diseases, FO-110 Tórshavn, Faroe Islands; debesc@hfs.fo; 4 Department of Immunology, Norwegian Veterinary Institute, 0454 Oslo, Norway; maria.dahle@vetinst.no

**Keywords:** PRV-1, *Piscine orthoreovirus*, HSMI, virulence, reassortment, viral evolution

## Abstract

Heart and skeletal muscle inflammation (HSMI) in farmed Atlantic salmon (*Salmo salar)* was first diagnosed in Norway in 1999. The disease is caused by *Piscine orthoreovirus*-1 (PRV-1). The virus is prevalent in farmed Atlantic salmon, but not always associated with disease. Phylogeny and sequence analyses of 31 PRV-1 genomes collected over a 30-year period from fish with or without HSMI, grouped the viral sequences into two main monophylogenetic clusters, one associated with HSMI and the other with low virulent PRV-1 isolates. A PRV-1 strain from Norway sampled in 1988, a decade before the emergence of HSMI, grouped with the low virulent HSMI cluster. The two distinct monophylogenetic clusters were particularly evident for segments S1 and M2. Only a limited number of amino acids were unique to the association with HSMI, and they all located to S1 and M2 encoded proteins. The observed co-evolution of the S1-M2 pair coincided in time with the emergence of HSMI in Norway, and may have evolved through accumulation of mutations and/or segment reassortment. Sequences of S1-M2 suggest selection of the HSMI associated pair, and that this segment pair has remained almost unchanged in Norwegian salmon aquaculture since 1997. PRV-1 strains from the North American Pacific Coast and Faroe Islands have not undergone this evolution, and are more closely related to the PRV-1 precursor strains not associated with clinical HSMI.

## 1. Introduction

Atlantic salmon (*Salmo salar*) aquaculture is a significant food production industry. Farmed fish are kept at high rearing densities, and outbreaks of infectious diseases strongly impact productivity and economic output [1]. Heart and skeletal muscle inflammation (HSMI) was reported for the first time in 1999, occurring during the seawater production phase in farmed Atlantic salmon in Mid-Norway [2], and rapidly emerged as an important disease. A few years later, disease outbreaks were reported from farms all along the Norwegian coast, and a maximum number of 181 outbreaks were registered in 2014, after which HSMI was taken off the national list of notifiable fish diseases. Since then, recordings of HSMI outbreaks have not been complete. In a questionnaire to fish health professionals in 2017, HSMI was judged as the most important viral disease in salmon farms from the northern and mid regions of Norway (severity score of 4.6 out of 5) [3]. HSMI is caused by *Piscine orthoreovirus* (PRV) [4,5], a virus which uses salmonid erythrocytes as main target cells and causes subsequent infection of myocytes and inflammation of heart and red skeletal muscles [6].

PRV belongs to the genus *Orthoreovirus* in the family Reoviridae, and has a non-enveloped double protein capsid containing a 10-segmented double-stranded RNA (dsRNA) genome. The genomic segments sort into three size groups; large (L1–L3), medium (M1–M3), and small (S1–S4) [4,7]. The genome encodes at least 11 proteins. Based on similarity with the well characterized mammalian orthoreovirus (MRV) and functional studies on PRV [5,7,8,9,10], eight proteins are structural components of the virus particle (inner capsid λ1, λ3, µ2, σ2, and outer capsid λ2, µ1, σ3, σ1) and three are non-structural (σNS, µNS, p13). The S1 and M2-encoded proteins σ3 and µ1 form a heterohexamer, (µ1)_3_(σ3)_3_ in the outer capsid [11]. In the structurally coupled σ3 and µ1 in MRV, mutations in the σ3 protein is linked to suppressor mutations in µ1 protein [12]. Similarly, in Vero-cell adapted MRV-3, σ1 and µ1 co-adaptation is linked to alterations in viral infection [13]. Proteolytic cleavage of σ3 and µ1 in the endosomes after endocytic viral uptake is important for entry and infectivity of reoviruses [14]. After entering the cellular cytoplasm, σ3 binds dsRNA, a function shown to modulate the host cell immune response [10]. The S1 segment also encodes p13, a non-fusogenic cytotoxic integral membrane protein [7,15]. In reoviruses, the replication of the dsRNA genome takes place after packaging of (+) ssRNA strands into the protein capsid. In case of an infection with two different genotypes of the reovirus in the same cell, this packaging may result in reassortants containing a mix of segments from the two viruses [16]. In addition, RNA viruses can genetically evolve through point mutations and recombination. In general, the mutation rate of RNA viruses is higher than in DNA viruses, and among RNA viruses, ssRNA viruses have a higher mutation rate than dsRNA viruses. The genome size, replication mode, and host factors influences the mutation rates in RNA viruses. The lower mutation rate of dsRNA viruses is likely due to their stamping machine mode of replication [17]. Reassortment can cause the emergence of strains with altered virulence and antigen properties, and have been linked to interspecies transmission [18]. 

Three subtypes of PRV, called PRV-1, -2 and -3, have been identified in salmonids. PRV-1 can cause HSMI in Atlantic salmon [5] and jaundice syndrome in Chinook salmon (*Oncorhynchus tshawytscha*) [19]. PRV-2 causes erythrocytic inclusion body syndrome (EIBS) in coho salmon (*Oncorhynchus kisutch*) [20] and PRV-3 causes pathological heart lesions in rainbow trout (*Oncorhynchus mykiss*) [21]. PRV-3 Germany was suggested as the likely causative agent of proliferative darkening syndrome (PDS) in brown trout (*Salmo trutta*) [22], but evidence lacked to conclude this [23]. The genome sequence of PRV-3 Germany is closely related to PRV-3 from coho salmon in Chile [22,24]. The species specificity is not absolute, and one genotype can infect several salmonid species. Pairwise nucleotide and amino acid sequence identities between PRV genotypes are 70%–90% [25], while within the PRV-1 genogroup, which is the most studied genogroup, the percent identity is in the upper 90’s [7]. Variability in the PRV-1 sequences, with a particular focus on the S1 segment, has been reported earlier and two genotypes have been proposed [26]. Genetic homogeneity was observed in the PRV-1 S1 sequences from a vast geographic range in the North American Pacific Coast (NAPC) over a 13-year period [27]. PRV-1 is reported to be widespread in Alaska and Washington state, and detected at 3.4% prevalence level in coho and Chinook salmon [28]. A higher prevalence was reported in Chinook salmon on the west coast of Vancouver Island exposed to aquaculture [29].

PRV-1 infection is reported to be common in wild returning salmon and sea trout in Norwegian rivers [30], but uncommon in landlocked salmon and brown trout [31]. Farmed Atlantic salmon in Norway became infected with PRV-1 a few months after transfer to seawater, but the infection may not necessarily manifest clinically [32]. During an outbreak, typical pathological lesions of HSMI can be found in the cardiac and skeletal muscles of most fish in the affected sea cage. However, the mortality is usually low, from insignificant to 20%, and influenced by many factors [2]. PRV infection is common in most countries with Atlantic salmon farming, like Chile [33], Canada [34], and Scotland [35], but some countries, like the Faroe Islands and Iceland, have not reported HSMI. PRV-1 is widespread in the NAPC region, and found in other Pacific salmon species like coho salmon, Chinook salmon, and sockeye salmon [28,36]. DiCicco et al. found HSMI histopathological lesions in farmed Atlantic salmon in British Columbia (BC), Canada, but the clinical signs were very mild with no elevated mortalities [37]. Recent findings from the same area point to mild heart inflammation [38], and failure to induce clinical HSMI in Atlantic salmon using PRV-containing material from BC, Canada [39]. This contrasts to the more severe pathological findings accompanied by increased mortalities and more than 100 annual outbreaks observed in Norway. The differences in disease manifestations could be due to differences in host, virus, environment, or a combination of these factors. In accordance with this, we have grouped BC strains as low virulent NAPC PRV group. The histopathological lesions of classical HSMI can be reproduced in experimental infections of Atlantic salmon using purified PRV particles from Norwegian isolates [5].

In this study, we obtained near-complete genome sequences of PRV-1 isolates collected over a 30-year period. Along with other available PRV-1 sequences from Atlantic salmon with or without a history of HSMI, phylogenetic and protein structure prediction analyses were used to explore the genetic diversity and evolutionary mechanisms of PRV-1 in farmed Atlantic salmon.

## 2. Materials and Methods

### 2.1. Virus Isolates

The coding regions were obtained by next generation sequencing technology from sea-reared Atlantic salmon from different sites of the Norwegian west coast sampled in 2005 (NOR-2005/TT), 2015 (NOR-2015/SSK), and 2015 (NOR-2015/MS). Two additional PRV genomes were retrieved from the Faroe Islands, FO/1978/15 from a smolt facility in 2015 and FO/41/16 from a sea site in 2016. Samples were plasma for NOR-2015/SSK and NOR-2015/MS, and heart and/or head kidney tissue in RNA later for the NOR-2005/TT and the Faroe samples. The sixth and seventh genomes were obtained from kidney samples from Atlantic salmon collected from sea sites in Norway in 1997 (NOR-1997) and 1988 (NOR-1988) stored at −80 °C, preceding the first description of HSMI with two and eleven years, respectively. The stored kidney samples were screened for the eventual presence of common viruses of Atlantic salmon by qPCR (i.e., infectious pancreatic necrosis virus, salmonid alphavirus, infectious salmon anemia virus, PRV-1, piscine myocarditis virus) and were only found positive for PRV-1. The kidney samples NOR-1988 and NOR-1997, were homogenized and injected in experimental Atlantic salmon at VESO Vikan in Norway, and replication of PRV-1 was demonstrated by qPCR in collected blood. The viral genome of NOR-1988 was retrieved from a fish collected at 4 wpc (PRV-1 Ct-value of 15.4 in blood pellet) and sequenced obtained by next generation sequencing (NGS) from the plasma sample. The viral genome of NOR-1997 was retrieved from a fish collected at 3 wpc (PRV-1 Ct-value of 16.2 in blood pellet) and Sanger sequenced obtained directly from the blood pellet. The challenge trial was approved by the Norwegian Animal Research Authority and performed in accordance with the recommendations of the current animal welfare regulations: FOR-1996-01-15-23 (Norway). All samples were from individual fish except for samples NOR-2005/TT and NOR-2015/SSK.

### 2.2. RNA Isolation

Tissues that had been stored in RNA later were added 650 µL QIAzol Lysis Reagent (Qiagen, Hilden, Germany) and plasma samples (250 µL) 750 µL Trizol LS (Life Technologies, Carlsbad, California, USA), as recommended by the manufacturers. Steel beads (5 mm) was added to samples and homogenized for 2 × 5 min at 25 Hz using TissueLyser II (Qiagen). After the addition of chloroform, the samples were centrifuged and the aqueous phase transferred to RNeasy Mini spin columns (Qiagen, Hilden, Germany). The remaining RNA purification procedure followed the manufacturer’s instructions. Total RNA was eluted in 50 μL RNase-free water, concentration determined using a NanoDrop ND-1000 spectrophotometer (ThermoFisher Scientific, Waltham, Massachusetts, USA), and subsequently stored at −80 °C.

### 2.3. RT-qPCR and Sanger Sequencing

The RT-qPCR was performed using the Quantitect Probe OneStep RT-PCR kit from Qiagen. For head kidney samples, the amount of total RNA was standardized to 100 ng per reaction (5 μL of 20 ng/uL). For plasma samples 5 μL total RNA was used in each reaction. Prior to one-step RT-qPCR, the template dsRNA was denatured at 95 °C for 5 min and cooled immediately on ice. The RT-qPCR targeted PRV gene segment S1 and was performed as previously described [40]. The coding regions of the revived sample from 1997 (NOR-1997) were amplified by PCR using primers shown in Table S1 and the PCR products were sequenced by Sanger sequencing (GATC Biotech AG, Konstanz, Germany).

### 2.4. NGS and Genome Assembly

The total RNA from the plasma, heart, and head kidney, stored at −80 °C, was added to 0.1× volume of 3 M sodium acetate (pH 7.5) and 2× volume of 100% ethanol, and mixed gently. Macrogen (Seoul, Korea) performed library preparation and sequencing of all samples except sample NOR-2005/TT. This genome was assembled using 454 sequencing data (GS FLX Titanium platform) from a previously published study [41]. For Illumina sequencing of NOR-2015/SSK, library preparation was performed using TruSeq RNA Library Prep v2 Kit (Illumina Inc., San Diego, CA, USA) and de novo sequencing performed on a MiSeq platform (2 × 300 PE). Libraries for samples NOR-2015/MS, NOR-1988, and the two samples from the Faroe Islands were also prepared using TruSeq RNA Library Prep v2 Kit without polyA enrichment or ribosomal depletion. DNaseI treatment was performed prior to library preparation. NOR-2015/MS and NOR-1988 were sequenced on a HiSeq2500 system (2 × 100 PE) and the two Faroe Island samples on a HiSeq2000 system (2 × 100 PE). Minor gaps in the sequences from the Faroe Islands and NOR/TT-2005 were closed using Sanger sequencing of RT-qPCR products (primers available upon request).

For the Illumina data, paired-end reads were assembled using a previously sequenced PRV genome as a reference [4] and the software BWA v0.6.2 [42]. Deviations from the reference sequence were scored and consensus sequences were generated using the software VarScan v2.3.9 [43]. Regions with low coverage and mutations deemed important were manually checked using the software BioEdit version 7.0.5 [44]. The 454 data were compared with the same reference PRV genome using BioEdit and assembly was performed manually. The final contigs were generated using AlignX software (Vector NTI Advance v11, ThermoFisher Scientific).

### 2.5. Phylogenetic Analyses

Multiple sequence alignments were performed using AlignX and MEGA X version 10.0.5 software (available from www.megasoftware.net) [45]. All phylogenetic trees were constructed using complete coding sequences from all the PRV genomes currently available in Genbank and the seven isolates sequenced in the present study. A separate phylogenetic analysis of segment S1 was performed including all available PRV-1 S1 sequences from GenBank spanning 830 nucleotides. Maximum likelihood (ML) was used together with the best-fitting nucleotide substitution model suggested by the MEGA X program [45]. Bootstrap values were calculated from 1000 replicates, and values above 70 were considered significant [46].

### 2.6. Protein Structure Analyses 

Protein secondary structure predictions were performed using PSIPRED v3.3, available at http://bioinf.cs.ucl.ac.uk/psipred/ [47]. A 3D-structure homology modeling of the σ3 proteins from the HSMI-associated NOR-2012 isolate was performed in order to spatially visualize the sequence differences between the two groups. The 3D model(s) of the full-length PRV1 σ3 protein was constructed using the threading method provided by the iTasser server, available online at https://zhanglab.ccmb.med.umich.edu/I-TASSER/ [48]. I-TASSER server identified MRV T3D σ3 protein (PDB ID:1FN9) as the most appropriate template (best fitting model) for 3D-homology modeling with the highest TM score of 0.862. Template modeling score (TM-score) measures the similarity of protein structures (score range 0-1). In general scores higher than 0.5 assume roughly the same fold. In order to be able to target relevant sequence information from the PRV-1 sequence database (i.e., Genbank and from present work) potentially linked to virulence, the software program QlikSense Desktop (https://www.qlik.com/us/products/qlik-sense) was used together with multiple sequence alignment of protein sequences. 

## 3. Results

### 3.1. Coding Sequences Obtained by Illumina and Sanger Sequencing

Here we present, in addition to previously published PRV-1 genomes from viruses originating from Norway, North American Pacific Coast (NAPC), and Chile, seven new isolates to the current PRV-1 genome sequence pool (Table 1). The 1997 (NOR-1997) and 1988 (NOR-1988) isolates were obtained from kidney samples from Atlantic salmon collected from sea sites in Norway and stored at –80°C, preceding the first description of HSMI with two and eleven years, respectively. Viral coding sequences were obtained from plasma following revival of these two isolates by injection of the stored frozen kidney homogenate in experimental fish. Illumina sequencing of NOR-2015/SSK, NOR-2015/MS, FO/1978/15, FO/41/16, and NOR-1988 provided full coverage of all coding regions (Table S2) except for FO/1978/15 and FO/41/16 from the Faroe Islands, where minor gaps were closed using traditional Sanger sequencing. Most of the NOR-2005/TT genome could be assembled from 454 sequencing, but overall coverage was poor with roughly 1000 PRV reads in total, and gaps were closed using Sanger sequencing. The coding sequences from NOR-1997 were obtained using Sanger sequencing only.

### 3.2. Phylogenetic Analyses of Individual PRV Genomic Segments Revealed Diversity

**Figure 1:**
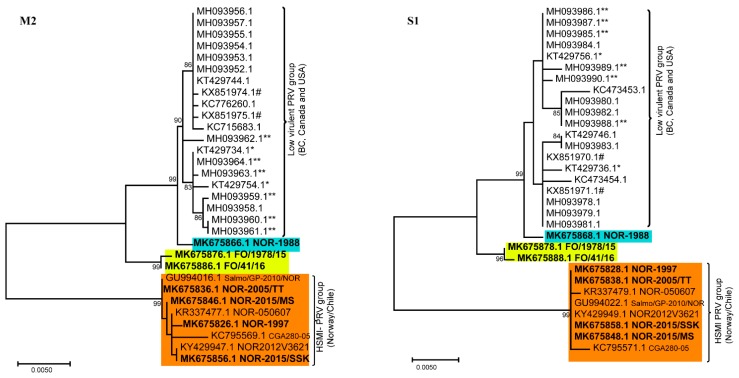
Phylogenetic trees constructed using M2 and S1 coding sequences from 31 PRV-1 isolates. For both gene segments, the Norwegian heart and skeletal muscle inflammation (HSMI) associated isolates, Chilean isolate, and Norwegian isolate from 1997, all group together forming a monophyletic cluster (orange background color). In contrast, the Norwegian isolate from 1988 (blue background color) and two Faroese isolates (yellow background color) group with the North American Pacific Coast isolates. Sequences obtained in the present study are shown in bold. * denotes isolates from coho and ** from Chinook salmon. # indicates PRV from BC HSMI longitudinal farm study

**Figure 2:**
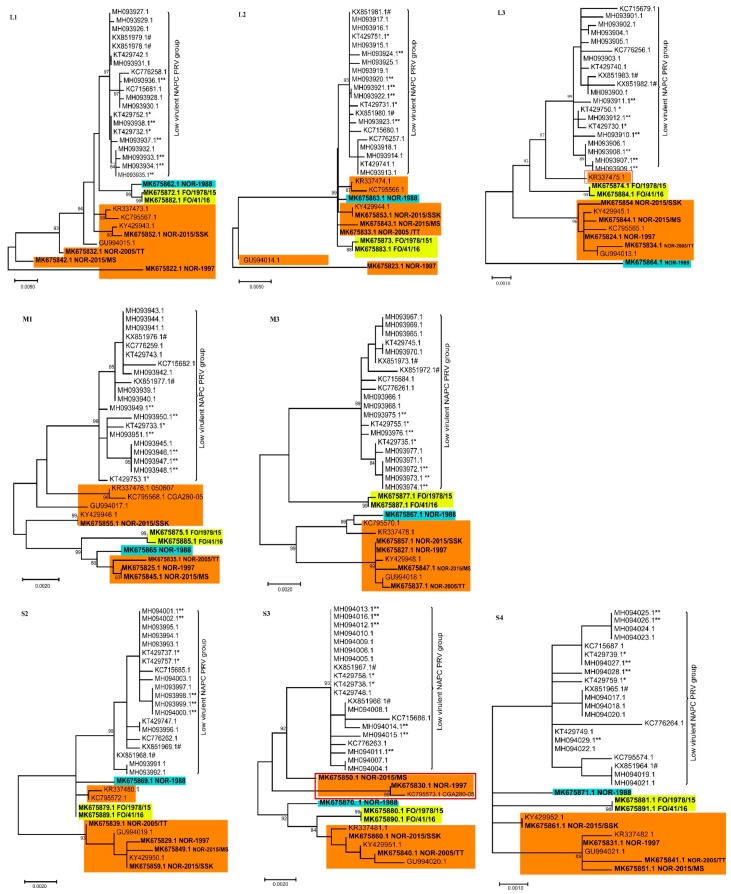
Phylogenetic trees constructed using coding sequences from segments L1, L2, L3, M1, M3, S2, S3, and S4 from 31 PRV-1 isolates. The North American Pacific Coast (NAPC) sequences consistently group together for all gene segments forming a monophyletic cluster. In contrast, the grouping of the Norwegian, Chilean, and Faroe Island isolates may vary from segment to segment. Color coding as in Figure 1 legend. Sequences obtained in the present study shown in bold. * denotes isolates from coho and ** from Chinook salmon. # indicates PRV from BC HSMI longitudinal farm study

Separate phylogenetic analyses of all ten genomic segments from the 31 available PRV-1 isolates revealed differential clustering depending on the segment for some isolates, with more consistent grouping patterns across the genome for other isolates. Using PRV-2 or PRV-3 as an outgroup did not change the grouping significantly, but collapsed the tree. Hence, to visualize minor differences within PRV-1 strains, the trees are presented as mid-point rooted. More specifically, PRV-1 from the NAPC grouped together in all ten gene segments with high bootstrap support, displaying lower intra-segment sequence diversity compared to the other PRV-1 isolates (Figure 1 and Figure 2). Hence, no segment reassortment could be observed within the isolates from NAPC. 

In the phylogenetic tree made from concatenated full genome amino acid sequences (Figure S1), Norwegian HSMI-associated isolates group separately from the monophyletic cluster generated by the NAPC and Faroe Islands.

The phylogenetic analyses showed that many HSMI isolates (orange color) changed group affiliation for the different genomic segments (Figure 1 and Figure 2), indicating possible reassortment events. The PRV-1 isolate from 1988 (highlighted in blue color) grouped with the HSMI-associated strains (orange color) for all segments except L1, S1, and M2, where it grouped with the NAPC and Faroese isolates (yellow color). The Faroese isolates also grouped with the NAPC PRV isolates for segments L3, M2, M3, and S1. The Norwegian 050607 isolate, collected from an HSMI outbreak in 2007, grouped together with other HSMI isolates except for L3 (orange box highlighted in red outline) where it grouped with the isolates from NAPC and Faroe Islands, with high bootstrap support (Figure 2). For segment M3, the Norwegian 050607 and the Chilean CGA280-05 isolates grouped with NOR-1988. Similarly, the HSMI associated NOR-2015/MS, NOR-1997, and Chilean CGA280-05 isolates changed group affiliation for segment S3 (orange box highlighted in red outline) (Figure 2). 

The bootstrap support for the two main monophyletic clusters generated for gene segments S1 and M2 were particularly high (Figure 1) and the two groups adhered completely to HSMI association or low-virulent HSMI association, the latter group constituted by the NAPC, Faroese, and NOR-1988 isolates. The number of nucleotide differences were high for segments S1 (30 nt) and M2 (60 nt) between the HSMI and other low virulent PRVs (NOR-1988, Faroes, NAPC isolates) (Table S4). In S1, this resulted in ten amino acid changes in σ3 and seven in p13. There were only three amino acid changes in the M2 encoded µ1. Most single nucleotide polymorphisms (SNPs) in the HSMI group were shared by all isolates within the group (i.e., 27/30 nt for S1, 51/60 nt for M2). The Faroese isolates, grouping slightly outside of the main NAPC cluster in S1 and M2, shared five and ten nucleotides, respectively, with the HSMI associated isolates. These shared nucleotides were not present in any of the NAPC isolates. Apart from this, sequence variation within the two main phylogenetic groups was very low for both segments. The high conservation of the S1 and M2 in the HSMI associated group was not found in the other segments.

### 3.3. None of the NAPC PRV-1 Isolates Group within the HSMI Clade

**Figure 3:**
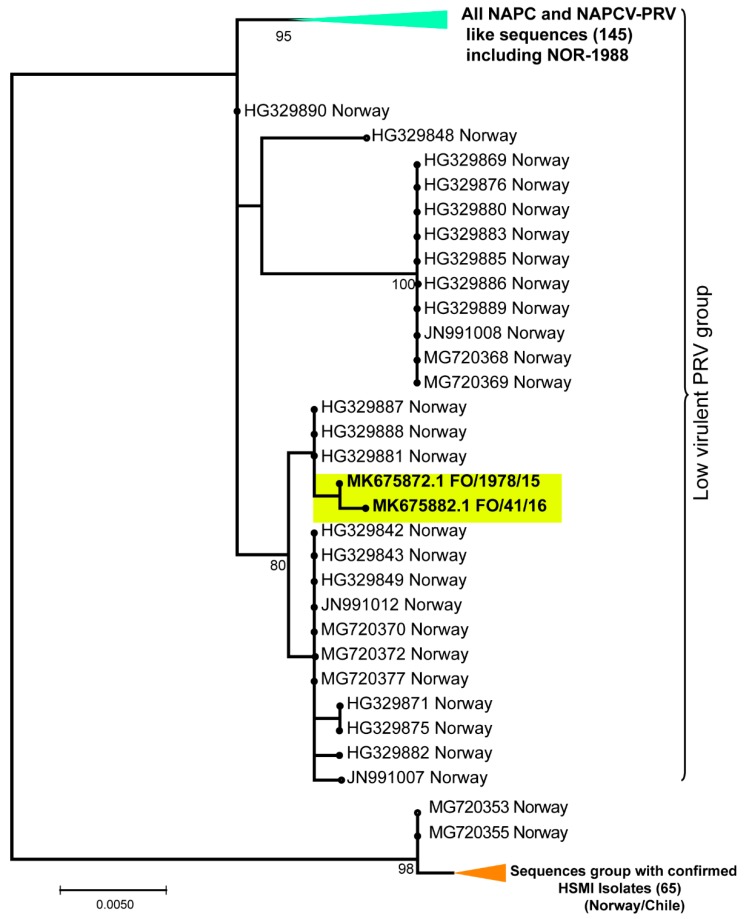
Phylogenetic trees constructed using partial S1 coding sequences (830 nt) of all available PRV-1 isolates in GenBank. The Faroese isolates are in the yellow box. HSMI associated isolates group separately from the NAPC sequences. Green triangle: All sequences from NAPC, and NAPC-like sequences, including NOR-1988. Orange triangle: All sequences from confirmed HSMI isolates.

When including all available partial coding sequences of minimum 830 nt in the phylogenetic analyses of segment S1 (240 sequences in all), they separated into two distinct monophyletic clusters (Figure 3). As for the tree generated with full–length S1 coding sequences from the 31 isolates (Figure 1), the HSMI associated and low virulent HSMI associated isolates grouped separately. All isolates in the HSMI associated group originated from Norway or Chile. Similarly, the S1 sequences from the non-HSMI associated NOR-1988 grouped with the NAPC and Faroese isolates. There were several S1 sequences originating from Norway that group together with the NAPC isolates (Figure 3). Interestingly, most of these NAPC-like sequences from Norway originated from samples collected from Atlantic salmon returning to rivers (wild or escaped) or from farmed salmon where no clinical information was available. Both phylogenetic analyses using complete (Figure 1) and partial (Figure 3) S1 coding sequences place the NAPC isolates within the low virulent group.

### 3.4. Amino Acid Residues Unique to HSMI Association are Located to Proteins Encoded by S1 and M2

Comparison of amino acid sequences of proteins encoded by the two main groups of PRV-1 isolates showed remarkable conservation except for σ3 and p13 (both encoded by S1) and µ1 (encoded by M2). No amino acid sites unique to the HSMI associated isolates were found for the other eight PRV-1 proteins (λ1, λ2, λ3, µ2, µNS, σ1, σ2, and σNS). In segment S1, the number of amino acid sites unique to HSMI association were ten for σ3 and seven for p13, most differences being amino acids with similar physiochemical properties (Table S3). Only two sites in the two overlapping reading frames encoding σ3 and p13 changed the amino acids in both proteins. The only other protein with amino acid sites unique to the HSMI associated isolates was the M2 encoded µ1 protein (three sites) (Table S3). 

**Figure 4:**
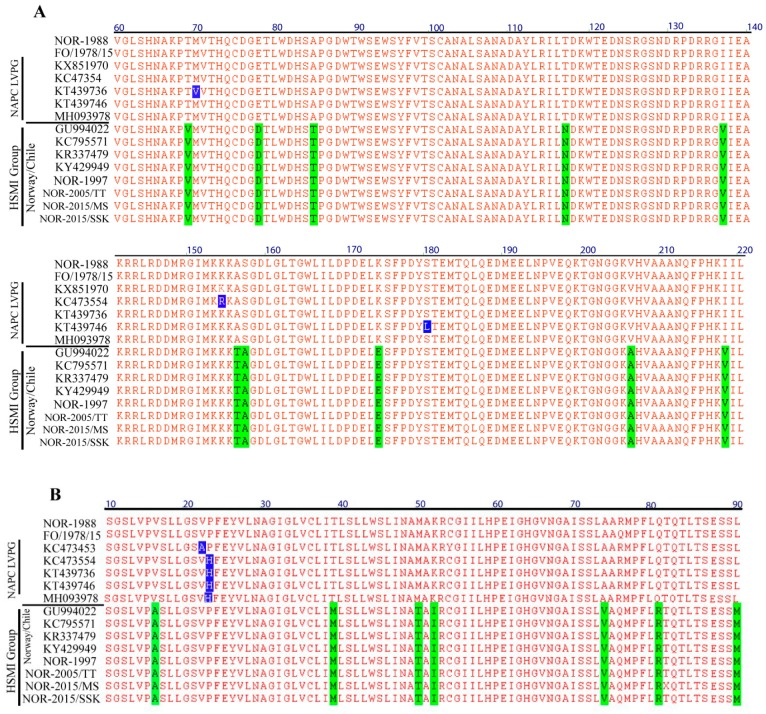
Sequence alignment of partial PRV-1 S1 encoded σ3 (**A**) and p13 proteins (**B**). For clarity, identical sequences in the low virulent group are not included in the alignment. Only sequence regions that contain variation among the isolates are presented. Amino acids unique to the HSMI associated group are shown with green background color and the amino acid differences in the low virulent group are shown with blue background color.

Finally, blast searches of all available complete and partial σ3 and µ1 sequences revealed that none of the unique amino acid sites in the HSMI associated group were present in available sequences from NAPC isolates. The NOR-1988 isolate, sampled from an apparently healthy fish eleven years prior to the first diagnosis of HSMI, is more similar to the NAPC and Faroese isolates for segments S1 and M2. In contrast, segments S1 and M2 from the NOR-1997 isolate sampled from a fish with unresolved disease two years before the first confirmed diagnosis of HSMI, were identical to the current Norwegian HSMI associated sequences. Within PRV isolates with HSMI association, the three proteins µ1, σ3, and p13 have identical amino acid sequences (Figure 4). The phylogenetic analysis of amino acid sequences encoded by the concatenated full PRV-1 genomes showed grouping of NAPC isolates together with NOR-1988 and Faroese isolates suggesting that these share a more recent common ancestor compared to HSMI isolates (Figure S1).

### 3.5. Predicted Differences between the HSMI Associated and Non-Associated Isolates Locate Mainly to Surface-Exposed Amino Acids in σ3 

**Figure 5:**
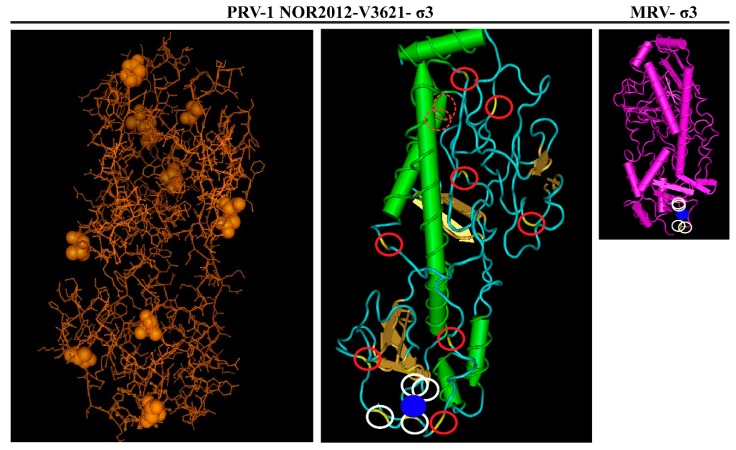
Visualization of the predicted 3D-structure of the NOR-2012 σ3 protein with Vector NTI 3D molecule viewer Cn3D v4.3 following structure homology modeling using the MRVσ3 protein (PDB ID:1FN9) as template, the most appropriate structure model to use as predicted by i-TASSER. The ten amino acid sites differing between the HSMI associated (here represented by NOR-2012) and low virulent HSMI isolates are indicated by or as yellow/brown balls (left picture) or as a yellow color enclosed by a red circle (middle picture). Amino acid side chains predicted to coordinate the Zn-ion, represented by a blue ball, are indicated by white circles. MRV-σ3 added for comparison.

Three-dimensional structure homology modeling of the σ3 proteins from the HSMI-associated NOR-2012 isolate was performed in order to spatially visualize the sequence differences between the two groups. I-TASSER server identified MRV T3D σ3 protein (PDB ID:1FN9) as the most appropriate template (best fitting model) for 3D-homology modeling with the highest TM score of 0.862. The modeled PRV-1 σ3 structure is similar to MRV σ3 with small and large lobes and a Zinc finger motif. Among the top five models predicted by I-TASSER, the model with the highest C score was used to visualize the protein. The amino acid sites differing between the HSMI associated and non-associated isolates seem to locate to surface exposed and more flexible coil regions (Figure 5). Only two of the ten sites locate to a predicted helical region.

Secondary structure predictions using PSI-blast based secondary structure prediction (PSIPRED) for the σ3 protein from HSMI and low virulent isolates showed very similar structures (Figure S2). Two of the ten amino acids that differed between the HSMI associated and non-associated groups located to a predicted sheet region and two other sites to a region containing a predicted helical structure.

## 4. Discussion

The analysis of 31 PRV-1 genomes collected from Atlantic salmon from Norway, the Faroes, NAPC, and Chile over a 30-year period included revived Norwegian isolates from samples collected in 1988 and 1997, pre-dating HSMI. The phylogenetic analyses revealed the presence of two distinct groups of PRV-1 sequences, which were either associated with highly virulent or low virulent strains. This grouping was particularly evident for genomic segments S1 and M2. For these two segments, nucleotide and amino acid sequences within the HSMI associated group showed remarkable conservation over the 20 years since the emergence of HSMI.

The first recorded HSMI outbreak was recorded in 1999 in Trøndelag, Mid-Norway [49], however, the S1 and M2 sequence identity of NOR-1997 isolate to the current HSMI outbreak viruses in Norway points to a possible presence of this disease before 1999. The NOR-1997 isolate was sampled from a disease outbreak with unresolved etiology. In contrast, sequence analysis of another pre-HSMI isolate, NOR-1988, showed high similarity to the low virulent HSMI group in segments L1, S1, and M2, but to the HSMI group for the other segments. Hence, PRV-1 was present in farmed Atlantic salmon well before the reported emergence of HSMI in Norway. Approximately 100 cases of HMSI outbreaks are recorded annually in farmed Atlantic salmon in Norway, but it is not a reportable disease, so the actual number of cases is likely much higher.

A recent study of Atlantic salmon experimentally challenged with a PRV-1 isolate from BC, found only minor signs of heart inflammation, which supported the phenotypic difference between NAPC and current Norwegian PRV-1 [38]. However, an experimental challenge study using different PRV strains could minimize the effect of host or environmental related factors in disease manifestation and identify putative virulence factors linked to HSMI. The presence of unique amino acid sites in HSMI associated PRV-1 isolates in the σ3, p13, and µ1 proteins suggest association of these proteins to the ability to induce HSMI, but the specific virulence determinants among σ3, p13, or µ1 proteins linked to HSMI need to be explored. The heterohexameric nature of (µ1)_3_(σ3)_3_ protein in the virus particle makes it likely that a gene linkage is involved for S1 and M2, indicating that only one of the proteins may be linked to HSMI, while the other could be forced to co-evolve. The high number of unique sites (i.e., ten for σ3 and seven for p13), in the two S1-encoded proteins suggest that these two proteins are central for the HSMI trait. Only two of the nucleotide differences change the amino acid sequence in both proteins (σ3, p13) on the bicistronic S1-segment, which suggest independent selection pressures.

The sequences of the S1 and M2 segments from Norwegian HSMI associated isolates differed significantly from the pre-HSMI isolate NOR-1988. One hypothesis for the emergence of HSMI in Norwegian Atlantic salmon farming is that the disease results from a reassortment event in PRV-1 introducing new S1 and M2 segments, followed by selection and spread of this S1/M2 pair. However, no donor strain of HSMI-associated S1/M2 pairs has been identified so far, and the number of available PRV sequences originating from before the emergence of HSMI is too limited to conclude about the molecular mechanisms involved in the evolution of S1 and M2. Mutational accumulation over time is an alternative hypothesis. There are few intermediate forms between the S1/M2 from HSMI associated and low virulent groups in the available selection of sequences. However, the Faroese isolates group slightly outside of the NAPC cluster in segments S1 and M2 sharing a number of nucleotides with the HSMI associated isolates that were not present in the NAPC isolates, suggesting that they might represent intermediate forms. Furthermore, the low mutation frequency, as indicated by completely conserved sequences of S1/ M2 from isolates associated with HSMI over the 20 years since the emergence of HSMI, also indicate that mutational accumulation should occur over a very long time. 

Reassortment events are common for mammalian *orthoreovirus* [50], and are indicated when genetic segments from the same isolate occupy different positions on phylogenetic trees of different segments [51], like we observed here. Some of the HSMI associated isolates grouped with the NOR-1988 for segments M3 and S3, indicating reassortment for these segments as well. Successful reassortment may result in progeny viruses more suited for selective constrains compared to parental viruses (i.e., increased viral fitness). We observed that segments S1 and M2 are genetically linked, which indicate that the structure and interaction of their encoded proteins are vital for virus fitness. For MRV, an in vitro forced reassortment event has been reported to alter virus infectivity and replication efficiency due to λ2 and σ1 protein mismatch [52]. 

The secondary and 3D structure predictions did not predict significant changes between the HSMI and low virulent associated strains’ σ3 proteins. The mostly synonymous substitutions were predicted to be surface exposed and located to apparently more disorganized regions of the protein. The minor changes in amino acid sequence in µ1 may represent an adaptation to the changes occurring in σ3 in order to maintain structural integrity of the (µ1)_3_(σ3)_3_ heterohexamer complex. It has been shown for MRV that a single amino acid change is sufficient to affect the interaction between µ1 and σ3 monomers and also the dsRNA binding ability of σ3 [53,54]. The dsRNA binding activity of MRV σ3 is an important inhibitor of the innate antiviral response, it inhibits both induction of type I interferon and activation of PKR [55]. Similarly, PRV σ3 also binds dsRNA, although no specific domain responsible for this binding has been determined [10]. The innate immune response is important for the onset of humoral and cellular acquired immunity. Cellular immunity is central in the pathogenesis of HSMI, which is characterized by the influx of CD8 lymphocytes in heart tissue [56]. An upregulation of genes related to innate antiviral response has been demonstrated repeatedly for experimental PRV-1 infections using PRV-1 isolates able to induce HSMI [5,6,57]. However, this was not found following experimental infection using a PRV-1 NAPC isolate that did not induce HSMI, and where the innate response, measured as an expression analysis of Mx, showed only modest upregulation [39]. This could indicate that an important difference between the HSMI-inducing and non-inducing groups of PRV-1 is the ability to induce innate antiviral response. The σ3 dsRNA binding affinity and thus its amino acid sequence could play a crucial role. 

The S1 encoded p13 has a more undefined function. p13 has been defined as an integral membrane protein located to intracellular membranes [15], further characterized as Golgi-like structures [7]. PRV p13 was also reported to be cytotoxic when overexpressed in non-salmonid cells [15], but the cytotoxic function has not been determined in vivo. Potentially, p13 could play a role in viral factory formation, viral particle release or modulation of antiviral responses. Regardless of its function, p13 is likely to be involved in molecular interaction with the host, which could be affected by changes in the amino acid sequence. A previous comparison between p13 sequences [27] did not reveal an association with HSMI. However, at that time, fewer PRV sequences were available.

This study suggests that the PRV-1 genotype causing HSMI in farmed Atlantic salmon has a recent common ancestor, and evolved prior to the rapid expansion of HSMI disease outbreaks in Norwegian Atlantic salmon farming which spread from a focal zone of outbreaks to all aquaculture areas within a few years after 1999 [2]. The HSMI-associated genotype contains unique S1 and M2 sequences, and the high conservation of the S1 and M2 sequences among the Norwegian HSMI outbreak isolates over the past 20 years strongly suggests an aquaculture-specific fitness improvement and higher virulence of PRV-1. The rapid selection and spread of this segment pair implies a relatively large evolutionary advantage for viruses harboring these versions of S1/M2 in the environment of industrialized, large-scale, farming of Atlantic salmon.

A low sequence diversity was observed among low virulent-associated NAPC S1 sequences over a period of 13 years indicating that these viruses are also well adapted to their host environment. Currently, the HSMI-associated PRV-1 variants have not been found in the NAPC region [27]. The phylogenetic analysis including the coding sequences from all segments from the 31 isolates, as well as the analysis of all available partial S1 sequences showed a distinct monophyletic clade for all sequences originating from the west coast of North America. The NAPC associated PRV-1 sequences are prevalent from other salmonid species including Chinook, coho, pink salmon, and rainbow trout. This low virulent PRV-1 group also encompassed some Chilean and Norwegian isolates like the pre-HSMI NOR-1988 isolate and viral sequences retrieved from wild fish in Norway [58]. Based on the S1 segment, the NAPC like sequences are still present in Norway and sequences are similar to either NOR-1988 or Faroese isolates. This indicates that both groups of sequences are present in Norway and Chile and are each evolutionarily stable.

## 5. Conclusions

The unique HSMI associated PRV-1 genotype is dominant in the current Norwegian and Chilean Atlantic salmon farming industry, while a low virulent PRV-1 group is reported from broad geographic regions and other salmonid species. Further full genome sequencing of PRV-1, including PRV-1 from wild Atlantic salmon, will provide more insights into the differential virulence evolution of PRV-1 in salmon.

## Figures and Tables

**Table 1:** *Piscine orthoreovirus*-1 (PRV-1) isolates sequenced in the study.

PRV Isolate	Associated Disease Status	Tissue Origin of Sequence	Accession Numbers
NOR-1988	Healthy	Plasma	MK675862–MK675871
NOR-1997	Unresolved, suspicion of ISA	Plasma	MK675822–MK675831
NOR-2005/TT	CMS	Heart	MK675832–MK675841
NOR-2015/SSK	HSMI	Plasma	MK675852–MK675861
NOR-2015/MS	HSMI-suspected	Plasma	MK675842–MK675851
FO/1978/15	Healthy	Head kidney	MK675872–MK675881
FO/41/16	Healthy	Head kidney	MK675882–MK675891

## Supplementary Materials

The following are available online at https://www.mdpi.com/1999-4915/11/5/465/s1, Figure S1: Phylogenetic analysis of concatenated full genome amino acid sequences (including p13); Figure S2: Secondary structure predictions of the σ3 and p13 proteins. Table S1: Primers used for Sanger sequencing of the NOR-1997 strain; Table S2: Number of reads of PRV-1 segments from the Illumina runs; Table S3: Amino acid differences between HSMI- and non-HSMI associated isolates compared to NOR-1988. Table S4. SNPs in M2 and S1 segment coding sequence of Norwegian HSMI strains

## References

[1] Ebert D., Bull J.J. (2003). Challenging the trade-off model for the evolution of virulence: Is virulence management feasible?. Trends Microbiol..

[2] Kongtorp R.T., Kjerstad A., Taksdal T., Guttvik A., Falk K. (2004). Heart and skeletal muscle inflammation in atlantic salmon, salmo salar l: A new infectious disease. J. Fish Dis..

[3] Hjeltnes B.W.C., Bang Jensen B., Haukaas A. (2018). The Fish Health Report 2017.

[4] Palacios G., Lovoll M., Tengs T., Hornig M., Hutchison S., Hui J., Kongtorp R.T., Savji N., Bussetti A.V., Solovyov A. (2010). Heart and skeletal muscle inflammation of farmed salmon is associated with infection with a novel reovirus. PLoS ONE.

[5] Wessel O., Braaen S., Alarcon M., Haatveit H., Roos N., Markussen T., Tengs T., Dahle M.K., Rimstad E. (2017). Infection with purified piscine orthoreovirus demonstrates a causal relationship with heart and skeletal muscle inflammation in atlantic salmon. PLoS ONE.

[6] Wessel O., Olsen C.M., Rimstad E., Dahle M.K. (2015). Piscine orthoreovirus (prv) replicates in atlantic salmon (salmo salar l.) erythrocytes ex vivo. Vet. Res..

[7] Markussen T., Dahle M.K., Tengs T., Lovoll M., Finstad O.W., Wiik-Nielsen C.R., Grove S., Lauksund S., Robertsen B., Rimstad E. (2013). Sequence analysis of the genome of piscine orthoreovirus (prv) associated with heart and skeletal muscle inflammation (hsmi) in atlantic salmon (salmo salar). PLoS ONE.

[8] Haatveit H.M., Nyman I.B., Markussen T., Wessel O., Dahle M.K., Rimstad E. (2016). The non-structural protein muns of piscine orthoreovirus (prv) forms viral factory-like structures. Vet. Res..

[9] Haatveit H.M., Wessel O., Markussen T., Lund M., Thiede B., Nyman I.B., Braaen S., Dahle M.K., Rimstad E. (2017). Viral protein kinetics of piscine orthoreovirus infection in atlantic salmon blood cells. Viruses.

[10] Wessel O., Nyman I.B., Markussen T., Dahle M.K., Rimstad E. (2015). Piscine orthoreovirus (prv) o3 protein binds dsrna. Virus Res..

[11] Dryden K.A., Wang G., Yeager M., Nibert M.L., Coombs K.M., Furlong D.B., Fields B.N., Baker T.S. (1993). Early steps in reovirus infection are associated with dramatic changes in supramolecular structure and protein conformation: Analysis of virions and subviral particles by cryoelectron microscopy and image reconstruction. J. Cell Biol..

[12] McPhillips T.H., Ramig R.F. (1984). Extragenic suppression of temperature-sensitive phenotype in reovirus: Mapping suppressor mutations. Virology.

[13] Sandekian V., Lemay G. (2015). Amino acids substitutions in sigma1 and mu1 outer capsid proteins of a vero cell-adapted mammalian orthoreovirus are required for optimal virus binding and disassembly. Virus Res..

[14] Nibert M.L., Fields B.N. (1992). A carboxy-terminal fragment of protein mu 1/mu 1c is present in infectious subvirion particles of mammalian reoviruses and is proposed to have a role in penetration. J. Virol..

[15] Key T., Read J., Nibert M.L., Duncan R. (2013). Piscine reovirus encodes a cytotoxic, non-fusogenic, integral membrane protein and previously unrecognized virion outer-capsid proteins. J. Gen. Virol..

[16] McDonald S.M., Nelson M.I., Turner P.E., Patton J.T. (2016). Reassortment in segmented rna viruses: Mechanisms and outcomes. Nat. Rev. Microbiol..

[17] Peyambari M., Warner S., Stoler N., Rainer D., Roossinck M.J. (2018). A 1000 year-old rna virus. J. Virol..

[18] Vijaykrishna D., Mukerji R., Smith G.J.D. (2015). Rna virus reassortment: An evolutionary mechanism for host jumps and immune evasion. PLoS Pathog..

[19] Di Cicco E., Ferguson H.W., Kaukinen K.H., Schulze A.D., Li S., Tabata A., Guenther O.P., Mordecai G., Suttle C.A., Miller K.M. (2018). The same strain of piscine orthoreovirus (prv-1) is involved in the development of different, but related, diseases in atlantic and pacific salmon in british columbia. Facets.

[20] Takano T., Nawata A., Sakai T., Matsuyama T., Ito T., Kurita J., Terashima S., Yasuike M., Nakamura Y., Fujiwara A. (2016). Full-genome sequencing and confirmation of the causative agent of erythrocytic inclusion body syndrome in coho salmon identifies a new type of piscine orthoreovirus. PLoS ONE.

[21] Hauge H., Vendramin N., Taksdal T., Olsen A.B., Wessel O., Mikkelsen S.S., Alencar A.L.F., Olesen N.J., Dahle M.K. (2017). Infection experiments with novel piscine orthoreovirus from rainbow trout (oncorhynchus mykiss) in salmonids. PLoS ONE.

[22] Kuehn R., Stoeckle B.C., Young M., Popp L., Taeubert J.E., Pfaffl M.W., Geist J. (2018). Identification of a piscine reovirus-related pathogen in proliferative darkening syndrome (pds) infected brown trout (salmo trutta fario) using a next-generation technology detection pipeline. PLoS ONE.

[23] Fux R., Arndt D., Langenmayer M.C., Schwaiger J., Ferling H., Fischer N., Indenbirken D., Grundhoff A., Dolken L., Adamek M. (2019). Piscine orthoreovirus 3 is not the causative pathogen of proliferative darkening syndrome (pds) of brown trout (salmo trutta fario). Viruses.

[24] Bohle H., Bustos P., Leiva L., Grothusen H., Navas E., Sandoval A., Bustamante F., Montecinos K., Gaete A., Mancilla M. (2018). First complete genome sequence of piscine orthoreovirus variant 3 infecting coho salmon (oncorhynchus kisutch) farmed in southern chile. Genome Announc..

[25] Dhamotharan K., Vendramin N., Markussen T., Wessel O., Cuenca A., Nyman I.B., Olsen A.B., Tengs T., Krudtaa Dahle M., Rimstad E. (2018). Molecular and antigenic characterization of piscine orthoreovirus (prv) from rainbow trout (oncorhynchus mykiss). Viruses.

[26] Kibenge M.J., Iwamoto T., Wang Y., Morton A., Godoy M.G., Kibenge F.S. (2013). Whole-genome analysis of piscine reovirus (prv) shows prv represents a new genus in family reoviridae and its genome segment s1 sequences group it into two separate sub-genotypes. Virol. J..

[27] Siah A., Morrison D.B., Fringuelli E., Savage P., Richmond Z., Johns R., Purcell M.K., Johnson S.C., Saksida S.M. (2015). Piscine reovirus: Genomic and molecular phylogenetic analysis from farmed and wild salmonids collected on the canada/us pacific coast. PLoS ONE.

[28] Purcell M.K., Powers R.L., Evered J., Kerwin J., Meyers T.R., Stewart B., Winton J.R. (2018). Molecular testing of adult pacific salmon and trout (oncorhynchus spp.) for several rna viruses demonstrates widespread distribution of piscine orthoreovirus in alaska and washington. J. Fish Dis..

[29] Tucker S., Li S.R., Kaukinen K.H., Patterson D.A., Miller K.M. (2018). Distinct seasonal infectious agent profiles in life-history variants of juvenile fraser river chinook salmon: An application of high-throughput genomic screening. PLoS ONE.

[30] Garseth A.H., Fritsvold C., Opheim M., Skjerve E., Biering E. (2013). Piscine reovirus (prv) in wild atlantic salmon, salmo salar l., and sea-trout, salmo trutta l., in norway. J. Fish Dis..

[31] Garseth A.H., Biering E. (2018). Little evidence to suggest salmonid freshwater reservoirs of piscine orthoreovirus (prv). J Fish Dis.

[32] Lovoll M., Alarcon M., Bang Jensen B., Taksdal T., Kristoffersen A.B., Tengs T. (2012). Quantification of piscine reovirus (prv) at different stages of atlantic salmon salmo salar production. Dis. Aquat. Org..

[33] Godoy M.G., Kibenge M.J.T., Wang Y., Suarez R., Leiva C., Vallejos F., Kibenge F.S.B. (2016). First description of clinical presentation of piscine orthoreovirus (prv) infections in salmonid aquaculture in chile and identification of a second genotype (genotype ii) of prv. Virol. J..

[34] Marty G.D., Morrison D.B., Bidulka J., Joseph T., Siah A. (2015). Piscine reovirus in wild and farmed salmonids in british columbia, canada: 1974-2013. J. Fish Dis..

[35] Ferguson H., Kongtorp R., Taksdal T., Graham D., Falk K. (2005). An outbreak of disease resembling heart and skeletal muscle inflammation in scottish farmed salmon, salmo salar l., with observations on myocardial regeneration. J. Fish Dis..

[36] Garver K.A., Marty G.D., Cockburn S.N., Richard J., Hawley L.M., Muller A., Thompson R.L., Purcell M.K., Saksida S. (2016). Piscine reovirus, but not jaundice syndrome, was transmissible to chinook salmon, oncorhynchus tshawytscha (walbaum), sockeye salmon, oncorhynchus nerka (walbaum), and atlantic salmon, salmo salar L.. J. Fish Dis..

[37] Di Cicco E., Ferguson H.W., Schulze A.D., Kaukinen K.H., Li S., Vanderstichel R., Wessel O., Rimstad E., Gardner I.A., Hammell K.L. (2017). Heart and skeletal muscle inflammation (hsmi) disease diagnosed on a british columbia salmon farm through a longitudinal farm study. PLoS ONE.

[38] Polinski M.P., Marty G.D., Snyman H.N., Garver K.A. (2019). Piscine orthoreovirus demonstrates high infectivity but low virulence in atlantic salmon of pacific canada. Sci. Rep..

[39] Garver K.A., Johnson S.C., Polinski M.P., Bradshaw J.C., Marty G.D., Snyman H.N., Morrison D.B., Richard J. (2016). Piscine orthoreovirus from western north america is transmissible to atlantic salmon and sockeye salmon but fails to cause heart and skeletal muscle inflammation. PLoS ONE.

[40] Finstad O.W., Dahle M.K., Lindholm T.H., Nyman I.B., Lovoll M., Wallace C., Olsen C.M., Storset A.K., Rimstad E. (2014). Piscine orthoreovirus (prv) infects atlantic salmon erythrocytes. Vet. Res..

[41] Lovoll M., Wiik-Nielsen J., Grove S., Wiik-Nielsen C.R., Kristoffersen A.B., Faller R., Poppe T., Jung J., Pedamallu C.S., Nederbragt A.J. (2010). A novel totivirus and piscine reovirus (prv) in atlantic salmon (salmo salar) with cardiomyopathy syndrome (cms). Virol. J..

[42] Li H., Durbin R. (2009). Fast and accurate short read alignment with burrows-wheeler transform. Bioinformatics.

[43] Koboldt D.C., Zhang Q., Larson D.E., Shen D., McLellan M.D., Lin L., Miller C.A., Mardis E.R., Ding L., Wilson R.K. (2012). Varscan 2: Somatic mutation and copy number alteration discovery in cancer by exome sequencing. Genome Res..

[44] Hall T.A. (1999). Bioedit: A User-Friendly Biological Sequence Alignment Editor and Analysis Program for Windows 95/98/nt. Nucleic Acids Symposium Series.

[45] Kumar S., Stecher G., Li M., Knyaz C., Tamura K. (2018). Mega x: Molecular evolutionary genetics analysis across computing platforms. Mol. Biol. Evol..

[46] Hillis D.M., Bull J.J. (1993). An empirical test of bootstrapping as a method for assessing confidence in phylogenetic analysis. Syst. Biol..

[47] Jones D.T. (1999). Protein secondary structure prediction based on position-specific scoring matrices. J. Mol. Biol..

[48] Roy A., Kucukural A., Zhang Y. (2010). I-tasser: A unified platform for automated protein structure and function prediction. Nat. Protoc..

[49] Kongtorp R.T., Taksdal T., Lyngøy A. (2004). Pathology of heart and skeletal muscle inflammation (hsmi) in farmed atlantic salmon salmo salar. Dis. Aquat. Org..

[50] Wenske E.A., Chanock S.J., Krata L., Fields B.N. (1985). Genetic reassortment of mammalian reoviruses in mice. J. Virol..

[51] Svinti V., Cotton J.A., McInerney J.O. (2013). New approaches for unravelling reassortment pathways. BMC Evol. Biol..

[52] Thete D., Danthi P. (2018). Protein mismatches caused by reassortment influence functions of the reovirus capsid. J. Virol..

[53] Mabrouk T., Lemay G. (1994). The sequence similarity of reovirus sigma-3 protein to picornaviral proteases is unrelated to its role in mu-1 viral protein cleavage. Virology.

[54] Bergeron J., Mabrouk T., Garzon S., Lemay G. (1998). Characterization of the thermosensitive ts453 reovirus mutant: Increased dsrna binding of sigma 3 protein correlates with interferon resistance. Virology.

[55] Imani F., Jacobs B.L. (1988). Inhibitory activity for the interferon-induced protein kinase is associated with the reovirus serotype 1 sigma 3 protein. Proc. Natl. Acad. Sci. USA.

[56] Mikalsen A.B., Haugland O., Rode M., Solbakk I.T., Evensen O. (2012). Atlantic salmon reovirus infection causes a cd8 t cell myocarditis in atlantic salmon (salmo salar l.). PLoS ONE.

[57] Dahle M.K., Wessel O., Timmerhaus G., Nyman I.B., Jorgensen S.M., Rimstad E., Krasnov A. (2015). Transcriptome analyses of atlantic salmon (salmo salar l.) erythrocytes infected with piscine orthoreovirus (prv). Fish Shellfish Immunol..

[58] Madhun A.S., Isachsen C.H., Omdal L.M., Einen A.C.B., Maehle S., Wennevik V., Niemela E., Svasand T., Karlsbakk E. (2018). Prevalence of piscine orthoreovirus and salmonid alphavirus in sea-caught returning adult atlantic salmon (salmo salar l.) in northern norway. J. Fish Dis..

