# Supplementary material for: Evolution of the Piscine orthoreovirus Genome Linked to Emergence of Heart and Skeletal Muscle Inflammation in Farmed Atlantic Salmon (Salmo salar)
Source: Viruses. 2019 May 22;11(5):465. doi: 10.3390/v11050465 (PMC6563308; doi:10.3390/v11050465)
Supplement: Supplementary file 1 [file viruses-11-00465-s001.pdf]

Supplementary Tables

Table S1 Primers used for Sanger sequencing of the NOR-1997 strain.

Primer name	*Nucleotide sequence (5′-3′)
Lambda1 UTR_Fw	GCCGCTCGAGTCTAGA**GATAATGTTTGTTTTGCCAT**
Lambda1 UTR_Rw	AAACGGGCCCTCTAGA**GATGAAGTTGTCATGTTTGT**
Lambda2 UTR_Fw	GCCGCTCGAGTCTAGA**GATAATTGTAACGACGAAAT**
Lambda2 UTR_Rw	AAACGGGCCCTCTAGA**GATGAAGAAGGAACGGCCTA**
Lambda3 UTR_Fw	GCCGCTCGAGTCTAGA**GATAATAATGGAGAAACCTA**
Lambda3 UTR_Rw	AAACGGGCCCTCTAGA**GATGAAGAAGGATCGGCCCA**
Mu2 UTR_Fw	GCCGCTCGAGTCTAGA**GATAATAACTCCTTTGCCAC**
Mu2 UTR_Rw	AAACGGGCCCTCTAGA**GATGAAAATCTCTTAAGCCC**
Mu1 UTR_Fw	GCCGCTCGAGTCTAGA**GATAAATTTGTTTAACAGGC**
Mu1 UTR_Rw	AAACGGGCCCTCTAGA**GATGAAGATTTGTCGTTCGG**
MuNS UTR_Fw	GCCGCTCGAGTCTAGA**GATAAAGCTTACGACACGTG**
MuNS UTR_Rw	AAACGGGCCCTCTAGA**GATGAGGAGGGGAGCTCACA**
Sigma1 UTR_Fw	GCCGCTCGAGTCTAGA**GATAAAGATCTTAACCGCAG**
Sigma1 UTR_Rw	AAACGGGCCCTCTAGA**GATGAAAAACAGGCTTACCG**
Sigma2 UTR_Fw	GCCGCTCGAGTCTAGA**GATAAATTTGTTGGTGACGA**
Sigma2 UTR_Rw	AAACGGGCCCTCTAGA**GATGAAGAGGCGTGCTGACT**
SigmaNS UTR_Fw	GCCGCTCGAGTCTAGA**GATAATTTTGATTGCATACA**
SigmaNS UTR_Rw	AAACGGGCCCTCTAGA**GATGAAGAGATGTTCGATTG**
Sigma3 UTR_Fw	GCCGCTCGAGTCTAGA**GATAAAGACTTCTGTACGTG**
Sigma3 UTR_Rw	AAACGGGCCCTCTAGA**GATGAATAAGACCTCCTTCC**

*PRV-1 sequence specific regions are shown in bold.

Table S2. Number of reads of PRV-1 segments from the Illumina HiSeq4000 run.

Segment	L1	L2	L3	M1	M2	M3	S1	S2	S3	S4
NOR-2015/SSK	3634	4460	2938	912	1016	1142	214	277	149	188
NOR-2015/MS	1047	5106	793	474	1106	505	837	690	924	933
FO/1978/15	207	270	163	120	87	98	128	52	76	40
FO/41/16	261	220	225	111	76	113	90	102	100	82
NOR-1988	211562	311365	203953	84962	72206	88916	55023	63314	72555	65535

Table S3. Amino acid differences between HSMI- and non-HSMI associated isolates compared to NOR-1988.

	***Non-HSMI isolates**			**HSMI Isolates**							
**Seg.**	**NOR-1988**	**FO/1978/15**	**All NAPC isolates**	**NOR-1997**	**NOR-2005/TT**	**NOR-050607**	**NOR-2015/SSK**	**NOR-2015/MS**	**NOR-V3621**	**GP-2010**	**CGA-280-05**
**L1**	$\mathbf{K_{16}}$									E_{16}	
	$\mathbf{A_{30}}$							T_{30}			
	$\mathbf{S_{60}}$										P_{60}
	$\mathbf{A_{63}}$	V_{63}	V_{63}	V_{63}	V_{63}	V_{63}	V_{63}	V_{63}	V_{63}	V_{63}	V_{63}
	$\mathbf{I_{179}}$			V_{179}	V_{179}			V_{179}			
	$\mathbf{V_{184}}$			A_{184}	A_{184}			A_{184}			
	$\mathbf{T_{200}}$							S_{200}			
	$\mathbf{D_{243}}$										G_{243}
	$\mathbf{E_{343}}$			G_{343}							
	$\mathbf{D_{347}}$			E_{347}							
	$\mathbf{N_{372}}$			D_{372}				D_{372}			
	$\mathbf{V_{490}}$			I_{490}							
	$\mathbf{I_{601}}$										T_{601}
	$\mathbf{N_{758}}$			S_{758}	S_{758}						
	$\mathbf{D_{937}}$			A_{937}				A_{937}			
	$\mathbf{V_{962}}$			I_{962}				I_{962}			
	$\mathbf{N_{996}}$			S_{996}							
	$\mathbf{R_{1065}}$										W_{1065}

	**V_{1266}**			I_{1266}	I_{1266}		I_{1266}	I_{1266}		I_{1266}	
**L2**	**A_{109}**			S_{109}						S_{109}	
	**D_{154}**			N_{154}							
	**G_{274}**										R_{274}
	**R_{412}**			K_{412}						K_{412}	
	**L_{434}**			F_{434}						F_{434}	
	**A_{447}**			T_{447}						T_{447}	
	**I_{529}**								L_{529}		
	**T_{932}**			A_{932}							
	**T_{1043}**			S_{1043}					A_{1043}		A_{1043}
	**V_{1055}**			I_{1055}							
	**F_{1066}**										L_{1066}
	**D_{1071}**			E_{1071}							
	**E_{1073}**										
	**I_{1075}**		V_{1075}								
	**P_{1141}**			L_{1141}							
	**I_{1257}**	T_{1257}									
**L3**	**F_{469}**										L_{469}
	**S_{594}**	A_{594}	A_{594}	A_{594}	A_{594}	A_{594}	A_{594}	A_{594}	A_{594}	A_{594}	A_{594}
	**F_{686}**										L_{686}
	**Q_{747}**				V_{747}						
	**V_{959}**				G_{959}						
	**T_{1031}**				S_{1031}						
**M1**	**R_{113}**	K_{113}							K_{113}		K_{113}
	**A_{511}**										T_{511}
	**K_{626}**								T_{626}		T_{626}
	**S_{710}**				A_{710}						
**M2**	**K_{113}**										E_{113}
	**I_{141}**	T_{141}									
	**S_{184}**			T_{184}	T_{184}	T_{184}	T_{184}	T_{184}	T_{184}	T_{184}	T_{184}
	**A_{262}**			S_{262}	S_{262}	S_{262}	S_{262}	S_{262}	S_{262}	S_{262}	S_{262}
	**N_{370}**			D_{370}	D_{370}	D_{370}	D_{370}	D_{370}	D_{370}	D_{370}	D_{370}
	**A_{389}**	V_{389}	V_{389}	V_{389}	V_{389}	V_{389}	V_{389}	V_{389}	V_{389}	V_{389}	V_{389}
	**E_{411}**										K_{411}
	**Q_{468}**										R_{468}
	**T_{487}**										A_{487}
**M3**	**L_{94}**	M_{94}	M_{94}	M_{94}	M_{94}	M_{94}	M_{94}	M_{94}		M_{94}	
	**T_{208}**							N_{208}			
	**V_{451}**	I_{451}	I_{451}	I_{451}	I_{451}	I_{451}	I_{451}	I_{451}		I_{451}	
	**A_{498}**		A_{498}						V_{498}		
	**T_{541}**	A_{541}	$A_{541\#}$	A_{541}	A_{541}	A_{541}	A_{541}	A_{541}	A_{541}	A_{541}	A_{541}
	**P_{563}**	S_{563}	S_{563}						P_{563}		
	**T_{653}**	A_{653}									
	**T_{658}**										A_{658}
**S1 (σ3)**	**N_{23}**										S_{23}

	**S$_{39}$**	P$_{39}$	P$_{39}$	P$_{39}$	P$_{39}$	P$_{39}$	P$_{39}$	P$_{39}$	P$_{39}$	P$_{39}$	P$_{39}$
	**T$_{69}$**			V$_{69}$	V$_{69}$	V$_{69}$	V$_{69}$	V$_{69}$	V$_{69}$	V$_{69}$	V$_{69}$
	**E$_{78}$**			D$_{78}$	D$_{78}$	D$_{78}$	D$_{78}$	D$_{78}$	D$_{78}$	D$_{78}$	D$_{78}$
	**A$_{85}$**			T$_{85}$	T$_{85}$	T$_{85}$	T$_{85}$	T$_{85}$	T$_{85}$	T$_{85}$	T$_{85}$
	**T$_{117}$**			N$_{117}$	N$_{117}$	N$_{117}$	N$_{117}$	N$_{117}$	N$_{117}$	N$_{117}$	N$_{117}$
	**I$_{137}$**			V$_{137}$	V$_{137}$	V$_{137}$	V$_{137}$	V$_{137}$	V$_{137}$	V$_{137}$	V$_{137}$
	**A$_{156}$**			T$_{156}$	T$_{156}$	T$_{156}$	T$_{156}$	T$_{156}$	T$_{156}$	T$_{156}$	T$_{156}$
	**S$_{157}$**			A$_{157}$	A$_{157}$	A$_{157}$	A$_{157}$	A$_{157}$	A$_{157}$	A$_{157}$	A$_{157}$
	**G$_{164}$**								G$_{164}$		
	**K$_{174}$**			E$_{174}$	E$_{174}$	E$_{174}$	E$_{174}$	E$_{174}$	E$_{174}$	E$_{174}$	E$_{174}$
	**V$_{206}$**			A$_{206}$	A$_{206}$	A$_{206}$	A$_{206}$	A$_{206}$	A$_{206}$	A$_{206}$	A$_{206}$
	**I$_{218}$**			V$_{218}$	V$_{218}$	V$_{218}$	V$_{218}$	V$_{218}$	V$_{218}$	V$_{218}$	V$_{218}$
**S1 (p13)**	**V$_{16}$**			A$_{16}$	A$_{16}$	A$_{16}$	A$_{16}$	A$_{16}$	A$_{16}$	A$_{16}$	A$_{16}$
	**T$_{39}$**			M$_{39}$	M$_{39}$	M$_{39}$	M$_{39}$	M$_{39}$	M$_{39}$	M$_{39}$	M$_{39}$
	**M$_{50}$**			T$_{50}$	T$_{50}$	T$_{50}$	T$_{50}$	T$_{50}$	T$_{50}$	T$_{50}$	T$_{50}$
	**K$_{52}$**			I$_{52}$	I$_{52}$	I$_{52}$	I$_{52}$	I$_{52}$	I$_{52}$	I$_{52}$	I$_{52}$
	**A$_{74}$**			V$_{74}$	V$_{74}$	V$_{74}$	V$_{74}$	V$_{74}$	V$_{74}$	V$_{74}$	V$_{74}$
	**R$_{76}$**	Q$_{76}$		Q$_{76}$	Q$_{76}$	Q$_{76}$	Q$_{76}$	Q$_{76}$	Q$_{76}$	Q$_{76}$	Q$_{76}$
	**Q$_{81}$**			R$_{81}$	R$_{81}$	R$_{81}$	R$_{81}$	R$_{81}$	R$_{81}$	R$_{81}$	R$_{81}$
	**L$_{91}$**			M$_{91}$	M$_{91}$	M$_{91}$	M$_{91}$	M$_{91}$	M$_{91}$	M$_{91}$	M$_{91}$
**S2**			**								
**S3**	**I$_{336}$**		**								V$_{336}$
**S4**	**A$_{75}$**										V$_{75}$
	**V$_{107}$**							A$_{107}$			
	**D$_{252}$**					N$_{252}$	N$_{252}$		N$_{252}$		N$_{252}$

*Amino acid sites consistently found in HSMI-associated isolates not present in NAPC, Faroe Island isolates and NOR-1988 are considered unique.

**The overall majority of NAPC sequences were identical to the NOR-1988 strain, but a few displayed minor differences.

Table S4. SNPs in M2 and S1 segment coding sequence of Norwegian HSMI strains compared to other NAPC and non-HSMI (NOR-1988, Faroes isolates).

S4. A

1. T80-C80	2. C189-T189	3. T203-C203 (only in CGA280-05)	4. C212-T212	5. A239-G239	6. G275-A275	7. A281-G281
8. C299-T299	9. G302-A302	10. A336-G336 (only in CGA280-05)	11. T362-C362	12. C404-G404	13. A410-G410	14. G419-A419
15. T464-C464	16. G550-C550	17. T581-C581 (In Salmo/GP-2010/NOR, NOR-2015/MS, CGA280-05)	18. C782-T782	19. G783-T783	20. T806-C806	21. T869-C869
22. C896-T896	23. C902-T902	24. C905-T905	25. G917-A917	26. T942-C942 (NOR-2015/MS, NOR-2015/SSK)	27. A944-G944	28. G950-A950
29. A960-C960	30. C983-T983	31. T1004-A1004	32. C1046-T1046	33. T1049-C1049	34. G1070-A1070	35. C1098-T1098
36. C1100-A1100	37. A1107-G1107	38. A1118-G1118 (only in NOR-2015/MS)	39. T1142-C1142	40. G1199-A1199	41. A1214-G1214	42. A1217-G1217
43. G1230-A1230 (only in NOR-2015/MS)	44. G1247-A1247	45. A1268-T1268	46. A1402-G1402 (only in NOR-2015/MS)	47. A1458-G1458 (only in NOR-2015/MS)	48. C1550-T1550	49. G1583-A1583
50. C1640-T1640	51. A1694-G1694	52. C1751-T1751	53. A1763-G1763	54. A1775-G1775	55. G1847-A1847	56. A1880-G1880
57. A1901-G1901	58. A1940-G1940	59. G1994-A1994	60. T2000-C2000 ((In NOR-1997, NOR-050607, NOR2012-V3621, NOR-2015/MS, NOR-			

			2015/SSK, CGA280-05)			

S4. B S1 segment

1. A68-G68 (only in CGA280-05)	2. C75-T75 (only in CGA280-05)	3. T126-C126	4. C195-T195	5. A205-G205	6. C206-T206	7. C228-T228
8. A234-T234	9. G253-A253	10. C300-T300	11. A321-G321	12. C350-A350	13. A409-G409	14. G466-A466
15. T469-G469	16. G491-A491 (NOR-050607)	17. T492-C492	18. G504-T504	19. C507-T507	20. A520-G520	21. G564-A564
22. T617-C617	23. A652-G652	24. A705-G705	25. T735-C735	26. A747-G747	27. A789-G789	28. C879-T879
29. C900-T900	30. T957-C957					

The non-HSMI (Faroes and NOR-1988) and other NAPC isolates nucleotides are indicated in black color with the position of nt starting from coding sequence

MH093978.1, MH093979.1, MH093980.1, MH093981.1, MH093982.1, MH093983.1, MH093984.1, MH093985.1, MH093986.1, MH093987.1, MH093988.1, MH093989.1, MH093990.1, MK675888.1 FO/41/16 MK675878.1 FO/1978/15, MK675868.1 NOR-1988, KX851971.1# KC473454.1, KT429736.1* , KX851970.1# KT429746.1, KC473453.1, KT429756.1*

The corresponding SNP in Norwegian HSMI isolates are highlighted in red color

KC795571.1 CGA280-05, MK675848.1 NOR-2015/MS, MK675858.1 NOR-2015/SSK, KY429949.1 NOR2012V3621, GU994022.1 Salmo/GP-2010/NOR, KR337479.1 NOR-050607, MK675838.1 NOR-2005/TT, MK675828.1 NOR-1997

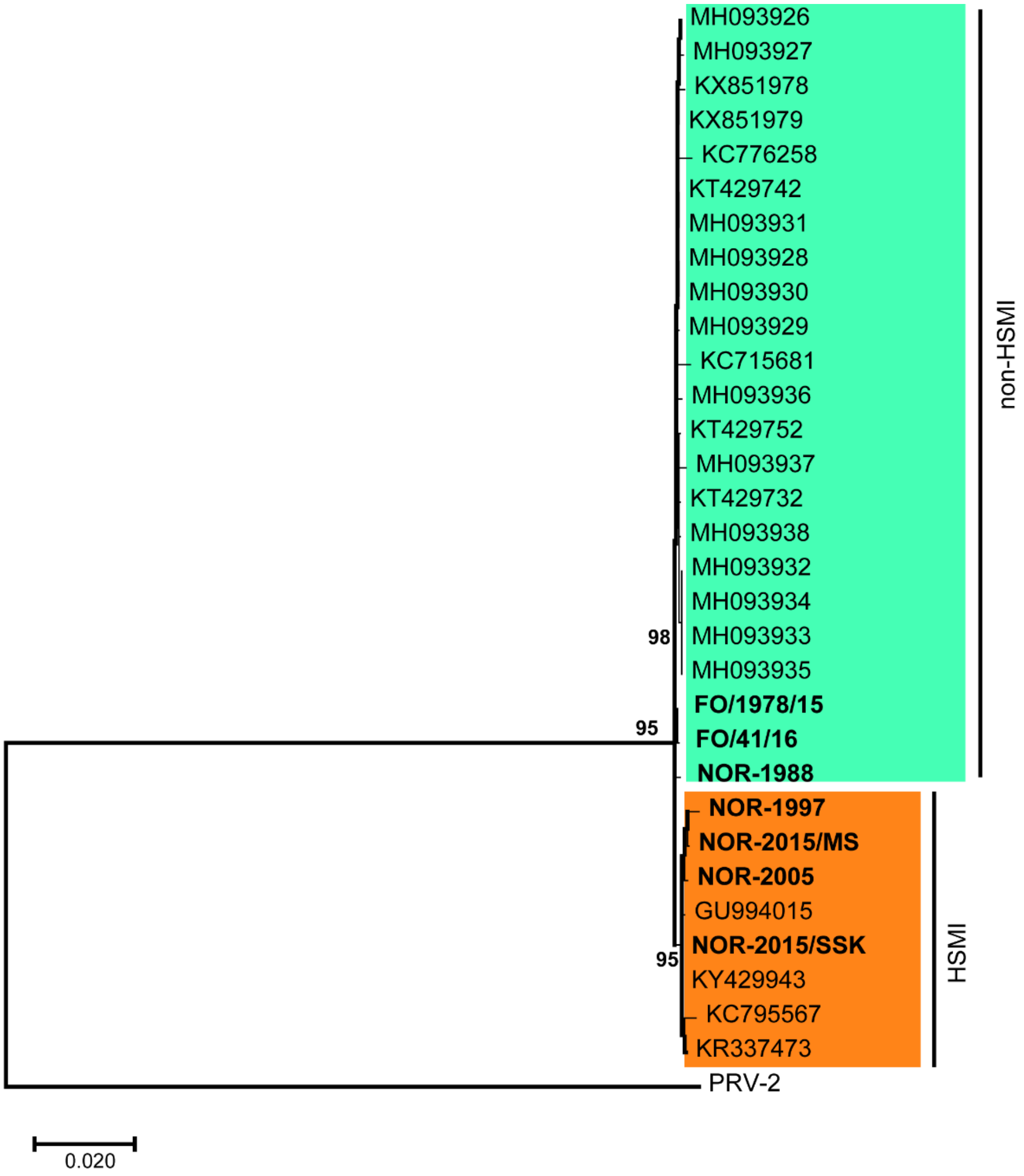

Fig. S1. Phylogenetic analysis of concatenated full genome amino acid sequences. PRV-2 is included as outgroup.

A. σ3 protein

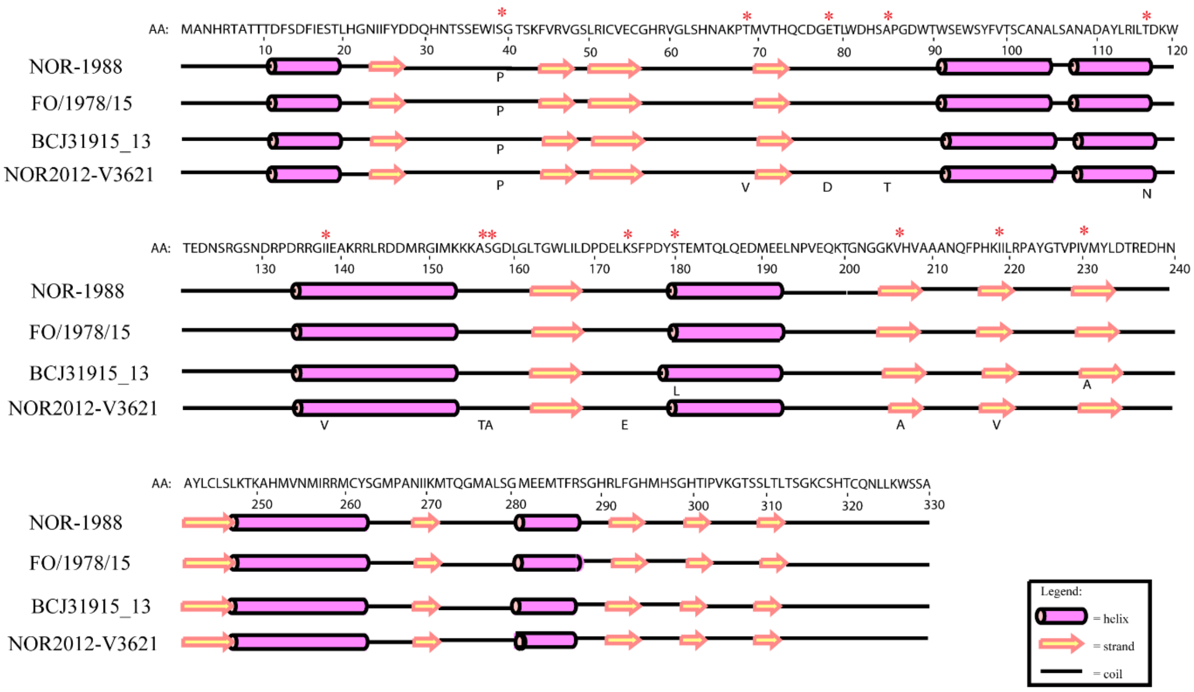

B. p13 protein

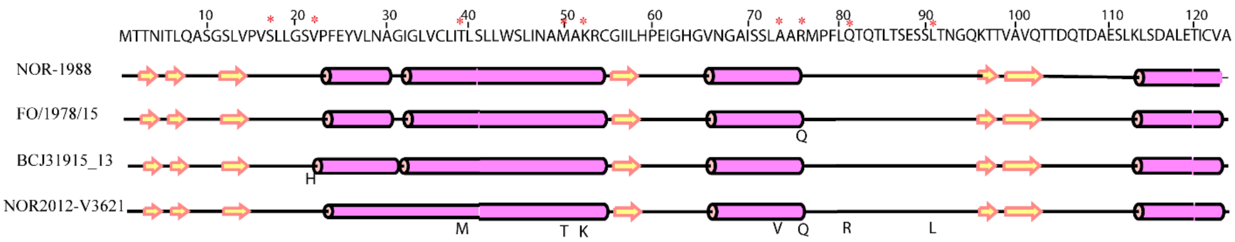

Fig. S2. Secondary structure predictions of σ3 and p13 proteins showing similar structure between NOR2012 and selected non-HSMI-associated strains for both proteins, respectively. Amino acid differences are indicated by red asterisks. The amino acid difference is indicated below the alignment.
